# Gene expression profiling in understanding the molecular pathogenesis of and response to canakinumab therapy in traps

**DOI:** 10.1186/1546-0096-12-S1-P138

**Published:** 2014-09-17

**Authors:** M Gattorno, R Torene, H Lachmann, L Obici, A Meini, V Tormey, R Caorsi, L Baeriswyl, U Affentranger, S Starck-Schwertz, M Letzkus, N Hartmann, K Abrams, N Nirmala

**Affiliations:** 1 G. Gaslini Institute, Genova, Italy; 2 Novartis Institutes for BioMedical Research (NIBR), Cambridge, Massachusetts, USA; 3 Royal Free Hospital, London, UK; 4 IRCCS Policlinico San Matteo, Pavia, Italy; 5 Spedali Civili, Brescia, Italy; 6 Galway University Hospitals, Galway, Ireland; 7 NIBR, Basel, Switzerland; 8 Novartis Pharmaceuticals Corporation, New Jersey, USA

## Introduction

TNF receptor-associated periodic syndrome (TRAPS) is an autoinflammatory disease causing unprovoked fevers, myalgia, abdominal pain, rash, headaches, and, in severe cases, AA amyloidosis. It is an autosomal dominant condition resulting from variants in the TNF super family receptor 1A (*TNFRSF1A*) gene [1]. A hallmark of TRAPS is a huge activation of the inflammatory response in the absence of autoantibodies or antigen-specific T-cells [1]. Canakinumab (CAN) is a high-affinity, fully human, selective, anti-IL-1β monoclonal antibody, developed for the treatment of autoinflammatory diseases [2].

## Objectives

To determine whether gene expression in whole blood can support a molecular mechanism for the activity of CAN in TRAPS patients (pts).

## Methods

Twenty pts with active TRAPS received open-label CAN 150mg sc/month for 4 months in an efficacy and safety study. These pts were assessed at Day 15 for response by physician global assessment (PGA) disease activity scale. Whole blood was collected at baseline, Day 15 and Day 113 from 19 of these pts and 1 sample each from 19 untreated age-matched healthy volunteers for analysis of gene expression levels by microarrays.

## Results

All 20 pts showed improvements in PGA score. Gene expression profiles of these pts were altered by treatment with CAN. Forty-six differentially expressed genes showed a >2-fold change after treatment with expression levels that shifted towards that of healthy volunteers. The disease-causing gene, *TNFRSF1A*,, drug target gene, *IL-1β*, and other inflammation related genes (e.g., *MAPK14*) were downregulated after treatment and several inflammation related pathways were evident among the differentially expressed genes. Many of the high confidence differentially expressed genes had expression levels that correlated with neutrophil count, however, neutrophil count alone could not account for the expression differences observed.

## Conclusion

Altogether, the gene expression data support a model in which CAN increases neutrophil apoptosis and reduces pro-inflammatory signals through its inhibition of IL-1β. CAN is able to reverse the overexpression of several genes associated with inflammatory response, including IL-1. Interestingly, IL-1 blockade normalized the overexpression of the disease-causing gene, *TNFRSF1A*, at the RNA level, suggesting a direct impact on the main pathogenic mechanism of TRAPS.

## Disclosure of interest

M. Gattorno: Grant / research support from: Novartis, SOBI. Speaker bureau of: Novartis, SOBI. R. Torene: Employee of: Novartis Institutes for BioMedical Research. H. Lachmann: Grant / research support from: Novartis, Celtic. Consultant for: Novartis. L. Obici: Consultant for: consultation fees from Pfizer. Speaker bureau of: speaker fees from Novartis Pharma. A. Meini: None declared. V. Tormey: None declared. R. Caorsi: None declared. L. Baeriswyl: Employee of: Novartis Institutes for BioMedical Research. U. Affentranger: Employee of: Novartis Institutes for BioMedical Research. S. Starck-Schwertz Employee of: Novartis Institutes for BioMedical Research. M. Letzkus: Employee of: Novartis Institutes for BioMedical Research. N. Hartmann Employee of: Novartis Institutes for BioMedical Research. K. Abrams: Shareholder of: Novartis, Employee of: Novartis Pharmaceuticals Corporation. N. Nirmala: Employee of: Novartis Institutes for BioMedical Research.
